# Real-Life Experience With Bictegravir/Emtricitabine/Tenofovir Alafenamide in Turkey

**DOI:** 10.7759/cureus.47253

**Published:** 2023-10-18

**Authors:** Umay Balcı, Ülkü Üser, Alper Tahmaz, Figen Sarigul Yildirim

**Affiliations:** 1 Infectious Diseases and Clinical Microbiology, Antalya Training and Research Hospital, Antalya, TUR; 2 Infectious Diseases and Clinical Microbiology, Akdeniz Sağlık Vakfı Yaşam Hospital, Antalya, TUR

**Keywords:** real-life experience, cd4 cell, virus, antiviral drug, hıv, bictegravir

## Abstract

Introduction: Single-tablet regimens (STRs) can increase treatment success and even improve the quality of life of human immunodeficiency virus (HIV) patients. In this study, we aim to analyze the real-life efficacy and tolerability data of people living with HIV (PLWH) initiated on or switched to bictegravir/emtricitabine/tenofovir alafenamide fumarate (BIC/FTC/TAF) as first-line treatment.

Materials and methods: This retrospective analysis was performed in HIV-1-positive patients who were initiated BIC/FTC/TAF in the HIV clinic between June 2020 and June 2022. Patients who received BIC/FTC/TAF for at least 12 months were included in this study. Virological suppression, laboratory parameters, side effects, and immunological response were analyzed at one, three, six, nine, and 12 months.

Results: A total of 116 patients, 66 (56.9%) treatment-experienced and 50 (43.1%) naive, were evaluated within the scope of the study. In the naive patient group, baseline HIV-RNA, CD4+ and CD8+ T cell counts, CD4/CD8 ratio, and estimated glomerular filtration rate (eGFR) values were significantly different in different follow-up months. The number of patients with HIV-1 RNA levels below 50 copies/mL was 55.9% in the first month, 73.7% in the third month, 90.2% in the sixth month, and 100% in the ninth and 12th months.

Conclusion: In our real-life observational study, BIC/FTC/TAF treatment achieved rapid viral suppression, maintained viral suppression in virally suppressed patients, and was effective for immunological recovery in both treatment-experienced and naive HIV patients. No serious side effects were observed. Our study has proved the potential of BIC/FTC/TAF as an important option in the treatment of HIV patients.

## Introduction

According to the Joint United Nations Programme on HIV/AIDS (UNAIDS), 39 million people in the world were living with human immunodeficiency virus (HIV) infection in 2022, and 630 thousand people died of acquired immunodeficiency syndrome (AIDS)-related illnesses. UNAIDS data show that today, 29.8 million of the 39 million people living with HIV (PLWH) globally are receiving life-saving treatment [1]. Today, HIV has become a chronic disease with antiretroviral therapy (ART). The initial ART for a person with HIV generally consists of two nucleoside analog reverse-transcriptase inhibitors (NRTIs), usually abacavir/lamivudine (ABC/3TC) or either tenofovir alafenamide/emtricitabine (TAF/FTC) or tenofovir disoproxil fumarate/emtricitabine (TDF/FTC), plus a drug from one of three drug classes: integrase strand transfer inhibitors (INSTIs), a non-nucleoside reverse transcriptase inhibitors (NNRTIs), or a protease inhibitor (PIs) [2].

Combination antiretroviral therapy (cART) for human immunodeficiency virus (HIV) infection provides viral suppression and immunological restoration. Since the number of pills was high in cART regimens, which were first introduced in 1996, side effects and non-adherence to treatment were also high in people living with HIV (PLWH) [3]. Reducing the number of tablets and providing single-tablet regimens (STRs) can improve treatment success and even improve quality of life by simplifying antiretroviral therapy and improving adherence [4,5].

The co-formulation of efavirenz/emtricitabine/tenofovir disoproxil fumarate (EFV/FTC/TDF) was the first daily STR [6]. There are currently five integrase strand transfer inhibitors (INSTI) co-formulations as STRs in Europe: bictegravir/emtricitabine/tenofovir alafenamide fumarate (BIC/FTC/TAF), dolutegravir/lamivudine/abacavir (DTG/3TC/ABC), elvitegravir/cobicistat/ emtricitabine/tenofovir alafenamide fumarate (EVG/c/FTC/TAF), dolutegravir/lamivudine (DTG/3TC), and dolutegravir/rilpivirine (DTG)/RPV) [7]. BIC/FTC/TAF, DTG/3TC/ABC, and EVG/c/FTC/TAF are available as STRs in Turkey. BIC/FTC/TAF was approved in Europe for the first time in 2018 and became available in Turkey in June 2020 for the treatment of HIV-1-infected adults with no history of resistance to tenofovir or emtricitabine and INSTI [8]. Clinical studies and meta-analyses have shown that BIC/FTC/TAF has high efficacy and has few side effects or drug-drug interactions [9,10]. Real-world data needs to be generated to assess the extent to which results from clinical trials are in line with the daily clinical practice.

A total of 29,284 adults were reported as being infected with HIV-1, and the proportion of ART patients was 79% by the end of 2020 in Turkey [11]. Virological success rates (76.6%-90%) are high in cases where the treatment is initiated. In 2020, 92% of HIV-positive patients in Turkey received INSTI-based treatment. INSTI drug class use will increase over time as they are included in the regimens recommended by the guidelines for first-line treatment in Turkey as well as in other countries. Among the reasons for the high rate of virological suppression in Turkey, the significant rate of treatment compliance could be due to the high use of STRs [12].

In this study, we aim to describe the characteristics of PLWH that were initiated on BIC/FTC/TAF as a first-line treatment or switched to BIC/FTC/TAF from other treatment options at our center in Turkey and analyze real-life efficacy and tolerability data among these patients.

## Materials and methods

Patients

This retrospective analysis was performed in HIV-1-positive patients who were initiated BIC/FTC/TAF (either as first-line therapy or as a switch from a previous regimen (TAF/FTC/EVG/c or TDF+FTC+DTG) at the HIV clinic in Antalya, Turkey, between June 2020 and June 2022. Patients older than 18 years of age, diagnosed with HIV, and receiving BIC/FTC/TAF for at least 12 months were included in this study. A total of 116 patients were included in the study; 50 patients were naive, and 66 patients had previous treatment experience.

Patient records were analyzed retrospectively. For the demographic data of the patients, age, gender, coinfection (hepatitis B and syphilis), comorbidities (hypertension, diabetes mellitus, liver disease, chronic kidney disease, chronic obstructive pulmonary disease, and heart disease/hyperlipidemia), baseline HIV-1 RNA, CD4+ T cell count, CD4/CD8 ratio, and regimens used in ART before switching were reported. Other laboratory parameters included baseline estimated glomerular filtration rate (eGFR), serum creatinine, hemoglobin, thrombocyte, glycosylated hemoglobin (HbA1C), total cholesterol (TC), high-density lipoprotein (HDL), low-density lipoprotein (LDL), triglyceride, and TC/HDL. These parameters were recorded at follow-up visits at one, three, six, nine, and 12 months. Data on previous ART, reasons for switching to BIC/FTC/TAF, discontinuation, and treatment modifications while on BIC/FTC/TAF were collected. Side effects occurring at the start of treatment or during a change of treatment and during the follow-up months were recorded. We also analyzed the virological suppression (HIV-1 RNA ≤ 50 copies/mL) and immunological response (CD4+ T cell count and CD4/CD8 ratio) at one, three, six, nine, and 12 months.

Ethical approval

Ethics committee approval was obtained at Health Sciences University Antalya Research and Training Hospital in Turkey in accordance with the 2008 Declaration of Helsinki (registration number: 14.10.2021 16/17).

Statistical analysis

Patient data collected within the scope of the study were analyzed using the IBM Statistical Package for the Social Sciences (SPSS) for Windows version 23.0 (IBM Corporation, Armonk, NY). Frequency and percentage are given for categorical data, and median and minimum and maximum descriptive values are reported for continuous data. The Wilcoxon test was used to determine the differences between the baseline measurements of each treatment group and the control measurements. The results were considered statistically significant when the p-value was less than 0.05.

## Results

A total of 116 patients, 66 (56.9%) treatment-experienced and 50 (43.1%) naive, were evaluated within the scope of the study. The average age of treatment-experienced or naive patients was 34 in both groups. Of the 116 patients, 102 (87.9%) were male. The demographic and clinical findings of the patients are given in Table 1. The age distribution of the patients was homogenous. While male gender and the comorbidity rate were higher in experienced patients compared to naive patients, the coinfection rate was found to be lower. It was found that 93.9% of experienced patients used EVG/c/FTC/TAF and 4.5% used DTG+TDF/FTC before BIC/FTC/TAF. Those who were on EVG/c/FTC/TAF were switched medications based on the recommendation of the physician after BIC/FTC/TAF was approved in Turkey. Among patients who used DTG+TDF/FTC, their treatment was switched to BIC/FTC/TAF because of itching in one patient, physician preference in one patient, and renal toxicity in one patient. Many baseline laboratory parameters of the experienced and naive patient group were found to have similar results. Four PLWH discontinued BIC/FTC/TAF treatment because one patient had a headache, two had a rash, and one was pregnant.

**Table 1:** Distribution of demographic and clinical findings of the patients COPD: chronic obstructive pulmonary disease, TAF/FTC/EVG/C: tenofovir alafenamide fumarate + emtricitabine elvitegravir+ cobicistat, TDF+FTC+DTG: tenofovir disoproxil fumarate + emtricitabine + dolutegravir, eGFR: estimated glomerular filtration rate, LDL: low-density lipoprotein, HDL: high-density lipoprotein, HIV: human immunodeficiency virus, HbA1C: glycosylated hemoglobin

Variables (N=116)	Experienced (n=66)	Naive (n=50)	Overall (n=116)
	Number (%) or median (minimum-maximum)	Number (%) or median (minimum-maximum)	Number (%) or median (minimum-maximum)
Age (years)	34 (18-68)	34 (19-62)	34 (18-68)
Gender (male)	61 (92.4)	41 (82)	102 (87.9)
Coinfection	6 (9.1)	7 (14)	13 (11.2)
Syphilis	4 (6.1)	7 (14)	11 (9.5)
Hepatitis B	2 (3)	0 (0)	2 (1.7)
Comorbidities	6 (9.1)	3 (6)	9 (7.8)
Hypertension	1 (1.5)	1 (2)	2 (1.7)
Diabetes mellitus	2 (3)	3 (6)	5 (4.3)
Liver disease	2 (3)	0 (0)	2 (1.7)
Chronic kidney disease	0 (0)	0 (0)	0 (0)
COPD	1 (1.5)	0 (0)	1 (0.9)
Heart disease/hyperlipidemia	2 (3)	1 (2)	3 (2.6)
Previous treatments			
TAF/FTC/EVG/c	62 (93.9)	0	62
TDF+FTC+DTG	3 (4.5)	0	3
Baseline HIV-RNA (copies/mL)	0 (0-124018)	28403.5 (0-10^9^)	0 (0-10^9^)
Baseline CD4 count (cells/μL)	861 (272-1806)	527 (42-1435)	702 (42-1806)
Baseline CD8 count (cells/μL)	966 (363-1855)	1033.5 (306-2886)	975 (306-2886)
Baseline CD4/CD8 ratio	0.8 (0.2-2.5)	0.6 (0.1-1.5)	0.7 (0.1-2.5)
Baseline eGFR (mL/minute)	90.5 (60-132)	100 (36-125)	92 (36-132)
Baseline creatinine (mg/dL)	1.1 (0.7-1.4)	1 (0.6-2.3)	1 (0.6-2.3)
Baseline hemoglobin (g/dL)	15.4 (11.4-17.2)	14.7 (10.2-17.6)	15 (10.2-17.6)
Baseline platelets (cells/mL)	252 (168-407)	229.5 (4.5-398)	245.5 (4.5-407)
Baseline HbA1C (%)	5.5 (5.2-107)	5.4 (4.6-10.6)	5.4 (4.6-107)
Baseline total cholesterol (mg/dL)	192.5 (165-298)	175 (114-351)	181 (114-351)
Baseline LDL (mg/dL)	119 (95-211)	101 (11-242)	105 (11-242)
Baseline HDL (mg/dL)	46.5 (34-74)	40.5 (24-193)	42.5 (24-193)
Baseline triglyceride (mg/dL)	112.5 (66-227)	127.5 (41-383)	123 (41-383)
Baseline total cholesterol/HDL ratio	4 (3.7-4.9)	4 (0.8-6.7)	4 (0.8-6.7)
Treatment change	3 (4.5)	1 (2)	4 (3.4)
Reason for treatment change			
Pregnancy	1 (33.3)	0	1 (25)
Adverse effects	2 (66.6)	1 (100)	3 (75)

The distribution of side effects observed in the patients included in the evaluation is given in Table 2. A total of 53 adverse events were observed in 31 (26.7%) patients in the first month of treatment; the most common side effects were weight gain with 12.1% (14 patients) and fatigue with 11.2% (13 patients). In the third month of the treatment, 24 side effects were observed in 22 (19%) patients; the most common side effects were weight gain with 8.6% (10 patients) and fatigue with 4.3% (five patients). In the sixth month of the treatment, 21 side effects were observed in 18 (15.5%) patients; the most common side effects were weight gain with 6.9% (eight patients) and urticaria/pruritus with 5.2% (six patients). In the ninth month of the treatment, 11 side effects were observed in 11 (9.5%) patients; the most common side effects were weight gain with 4.3% (five patients) and weakness and urticaria/itching with 1.7% (two patients). No side effects were observed in the first year of treatment.

**Table 2:** Distribution of adverse effects in the patients during follow-up

Adverse effects	Month 1 (number (%))	Month 3 (number (%))	Month 6 (number (%))	Month 9 (number (%))	Month 12 (number (%))
Weakness	13 (11.2)	5 (4.3)	4 (3.4)	2 (1.7)	0 (0)
Nausea	1 (0.9)	0 (0)	0 (0)	0 (0)	0 (0)
Headache	3 (2.6)	0 (0)	0 (0)	0 (0)	0 (0)
Diarrhea	2 (1.7)	1 (0.9)	1 (0.9)	1 (0.9)	0 (0)
Dyspepsia	2 (1.7)	1 (0.9)	0 (0)	1 (0.9)	0 (0)
Urticaria/itching	8 (6.9)	4 (3.4)	6 (5.2)	2 (1.7)	0 (0)
Joint pain	6 (5.2)	1 (0.9)	1 (0.9)	0 (0)	0 (0)
Weight gain	14 (12.1)	10 (8.6)	8 (6.9)	5 (4.3)	0 (0)
Weight loss	2 (1.7)	1 (0.9)	0 (0)	0 (0)	0 (0)
Hair loss	2 (1.7)	1 (0.9)	1 (0.9)	0 (0)	0 (0)
Total	53	24	21	11	0

**Figure 1:**
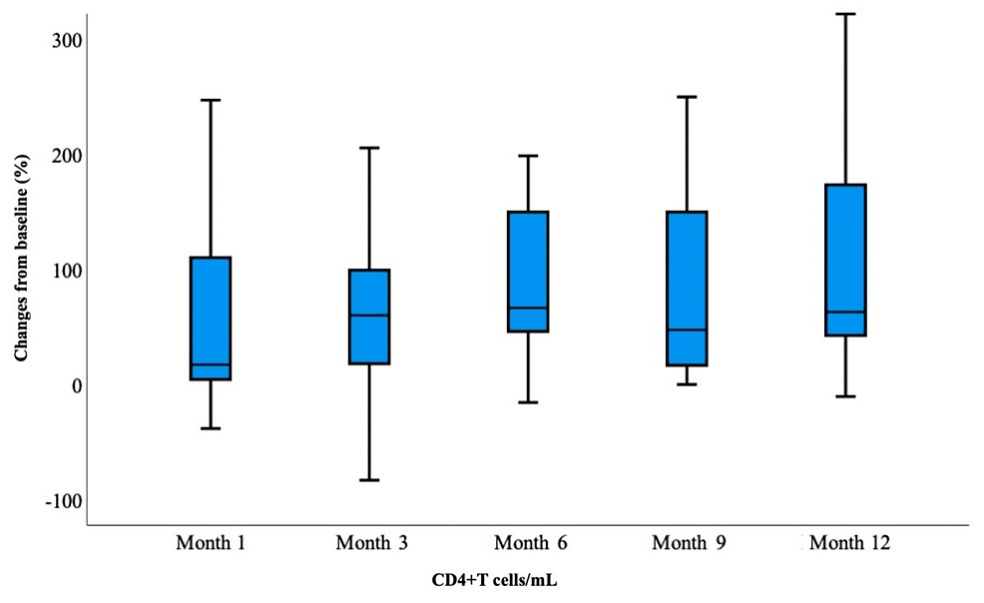
Treatment-experienced patient group: CD4+ T cells/mL at baseline and follow-up at one, three, six, nine, and 12 months

**Figure 2:**
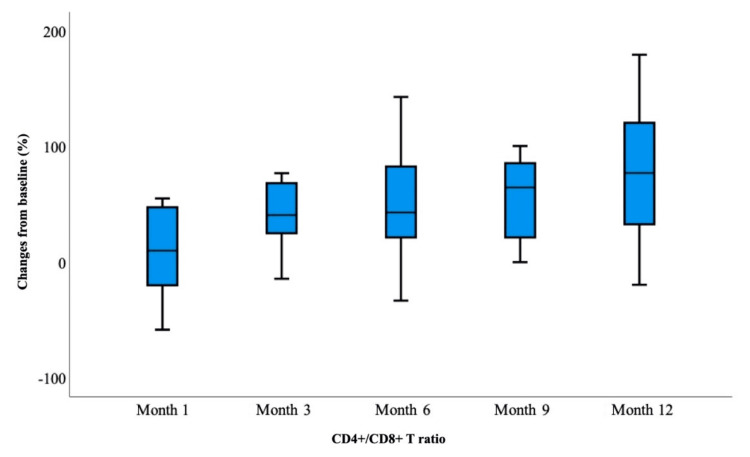
Treatment-naive patient group: CD4+/CD8+ ratio at baseline and follow-up at one, three, six, nine, and 12 months

**Figure 3:**
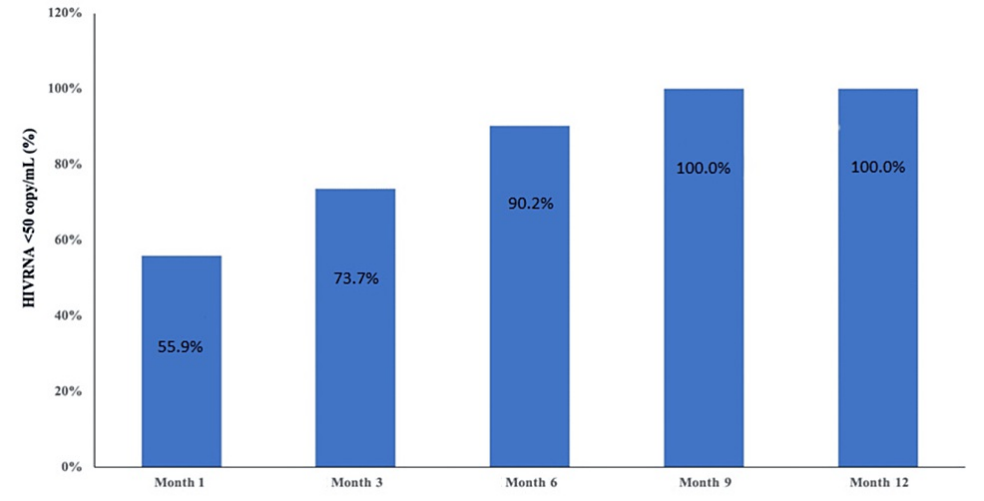
Treatment-naive patient group: HIV-RNA at baseline and follow-up at one, three, six, nine, and 12 months

Table 3 shows the baseline laboratory parameters of treatment-experienced and naive patients and the distribution of laboratory parameters in the first, third, sixth, ninth, and 12th months of their treatment. There was no statistically significant difference between the baseline measurement values in each laboratory parameter of the treatment-experienced patient group and the measurement values in the follow-up months (p>0.05). In the treatment-experienced patient group, although not significant, a decrease in CD4+ T cell count was observed in the first and third months (Figure 1). In the naive patient group, baseline HIV-RNA, CD4+ and CD8+ T cell counts, CD4/CD8 ratio (Figure 2), and eGFR values were found to be statistically significantly different at different follow-up months. HIV-RNA value became <50 copies/mL from the first month and was statistically significant at each follow-up according to baseline measurement (p<0.05). The number of patients with HIV-1 RNA levels below 50 copies/mL was 55.9% in the first month, 73.7% in the third month, 90.2% in the sixth month, and 100% in the ninth and 12th months (Figure 3).

**Table 3:** Distribution of laboratory parameters of treatment-experienced and naive patients ^a^Baseline versus first month ^b^Baseline versus third month ^c^Baseline versus sixth month ^d^Baseline versus ninth month ^e^Baseline versus 12th month HIV: human immunodeficiency virus, eGFR: estimated glomerular filtration rate, LDL: low-density lipoprotein, HDL: high-density lipoprotein

Laboratory parameters	Experienced		Naive	
	Median (minimum-maximum)	p-value	Median (minimum-maximum)	p-value
Baseline HIV-RNA (copies/mL)	0 (0-124018)	-	28403.5 (0-10^9^)	-
Month 1 HIV-RNA (copies/mL)	0 (0-61)	^a^0.109	0 (0-23985)	^a^<0.001
Month 3 HIV-RNA (copies/mL)	0 (0-112)	^b^0.208	0 (0-250)	^b^<0.001
Month 6 HIV-RNA (copies/mL)	0 (0-6593)	^c^0.712	0 (0-12841)	^c^<0.001
Month 9 HIV-RNA (copies/mL)	0 (0-6593)	^d^0.917	0 (0-0)	^d^<0.001
Month 12 HIV-RNA (copies/mL)	0 (0-64)	^e^0.143	0 (0-42)	^e^<0.001
Baseline CD4 count (cells/μL)	861 (272-1806)	-	527 (42-1435)	-
Month 1 CD4 count (cells/μL)	778 (290-1368)	^a^0.820	651 (250-1430)	^a^0.006
Month 3 CD4 count (cells/μL)	750 (312-2115)	^b^0.904	667.5 (112-1296)	^b^0.003
Month 6 CD4 count (cells/μL)	836 (240-1558)	^c^0.956	728 (225-1674)	^c^<0.001
Month 9 CD4 count (cells/μL)	924 (272-1558)	^d^0.326	703 (220-1188)	^d^0.013
Month 12 CD4 count (cells/μL)	882 (210-1568)	^e^0.934	754 (330-1260)	^e^0.004
Baseline CD8 count (cells/μL)	966 (363-1855)	-	1033.5 (306-2886)	-
Month 1 CD8 count (cells/μL)	989 (480-1760)	^a^0.532	1026 (504-2516)	^a^0.922
Month 3 CD8 count (cells/μL)	980 (420-1927)	^b^0.170	827 (420-1953)	^b^0.041
Month 6 CD8 count (cells/μL)	903 (276-1692)	^c^0.088	977.5 (340-2205)	^c^0.091
Month 9 CD8 count (cells/μL)	930 (480-1800)	^d^0.253	846 (442-1488)	^d^0.048
Month 12 CD8 count (cells/μL)	900 (405-2059)	^e^0.940	870 (456-3550)	^e^0.404
Baseline CD4/CD8 ratio	0.8 (0.2-2.5)	-	0.6 (0.1-1.5)	-
Month 1 CD4/CD8 ratio	0.8 (0.5-1.4)	^a^0.334	0.6 (0.1-2.2)	^a^0.013
Month 3 CD4/CD8 ratio	0.8 (0.4-2.1)	^b^0.265	0.8 (0.2-2.6)	^b^<0.001
Month 6 CD4/CD8 ratio	0.9 (0.2-2.4)	^c^0.299	0.8 (0.2-2.7)	^c^<0.001
Month 9 CD4/CD8 ratio	0.9 (0.3-1.8)	^d^0.211	0.9 (0.3-1.5)	^d^0.001
Month 12 CD4/CD8 ratio	0.9 (0.2-2.1)	^e^0.814	0.8 (0.2-1.6)	^e^0.004
Baseline eGFR (mL/minute)	90.5 (60-132)	-	100 (36-125)	-
Month 1 eGFR (mL/minute)	84 (42-121)	^a^0.195	87 (30-122)	^a^<0.001
Month 3 eGFR (mL/minute)	87 (54-127)	^b^0.170	95.5 (39-99.5)	^b^0.026
Month 6 eGFR (mL/minute)	90 (63-123)	^c^0.052	90.5 (66-126)	^c^<0.001
Month 9 eGFR (mL/minute)	90 (62-121)	^d^0.058	93 (30-112)	^d^<0.001
Month 12 eGFR (mL/minute)	86 (68-119)	^e^0.052	85 (62-104)	^e^0.001
Baseline total cholesterol (mg/dL)	192.5 (165-298)	-	175 (114-351)	-
Month 1 total cholesterol (mg/dL)	NA	-	NA	-
Month 3 total cholesterol (mg/dL)	207 (157-292)	^b^0.285	179 (140-221)	^b^0.465
Month 6 total cholesterol (mg/dL)	184 (113-250)	^c^0.180	216 (155-256)	^c^0.655
Month 9 total cholesterol (mg/dL)	190.5 (156-225)	^d^0.855	180 (107-230)	^d^0.279
Month 12 total cholesterol (mg/dL)	176 (126-241)	^e^0.655	180 (142-253)	^e^0.173
Baseline LDL (mg/dL)	119 (95-211)	-	101 (11-242)	-
Month 1 LDL (mg/dL)	NA	-	NA	-
Month 3 LDL (mg/dL)	134 (88-400)	^b^0.285	97 (73-142)	^b^0.713
Month 6 LDL (mg/dL)	105 (50-165)	^c^0.655	132 (104-145)	^c^0.655
Month 9 LDL (mg/dL)	121 (91-151)	^d^0.940	96 (60-150)	^d^0.465
Month 12 LDL (mg/dL)	92 (68-159)	^e^0.180	127 (91-167)	^e^0.112
Baseline HDL (mg/dL)	46.5 (34-74)	-	40.5 (24-193)	-
Month 1 HDL (mg/dL)	NA	-	NA	-
Month 3 HDL (mg/dL)	54 (39-66)	^b^0.285	57 (38-73)	^b^0.144
Month 6 HDL (mg/dL)	53 (33-85)	^c^0.180	37 (33-59)	^c^0.655
Month 9 HDL (mg/dL)	55 (55-55)	^d^0.190	37 (30-44)	^d^0.581
Month 12 HDL (mg/dL)	47 (34-94)	^e^0.655	47 (32-61)	^e^0.686
Baseline triglyceride (mg/dL)	112.5 (66-227)	-	127.5 (41-383)	-
Month 1 triglyceride (mg/dL)	NA	-	NA	-
Month 3 triglyceride (mg/dL)	99 (48-488)	^b^0.109	99 (49-128)	^b^0.715
Month 6 triglyceride (mg/dL)	94 (46-207)	^c^0.317	235 (90-258)	^c^0.655
Month 9 triglyceride (mg/dL)	71 (48-94)	^d^0.090	110 (53-329)	^d^0.500
Month 12 triglyceride (mg/dL)	170 (37-394)	^e^0.180	113.5 (81-146)	^e^0.686
Baseline total cholesterol/HDL ratio	4 (3.7-4.9)	-	4 (0.8-6.7)	-
Month 1 total cholesterol/HDL ratio	NA	-	NA	-
Month 3 total cholesterol/HDL ratio	4.0 (2.8-6.1)	^b^0.109	2.9 (2.5-4.6)	^b^0.144
Month 6 total cholesterol/HDL ratio	3.4 (2.5-4.5)	^c^0.655	4.7 (4.3-5.8)	^c^0.655
Month 9 total cholesterol/HDL ratio	3.5 (2.8-4.1)	^d^0.640	4.2 (3.5-6.6)	^d^0.893
Month 12 total cholesterol/HDL ratio	3.7 (2.4-5.1)	^e^0.180	4.3 (3.5-4.8)	^e^0.345

The CD4+ T cell count tended to increase in the follow-up months compared to the baseline measurement, and the measurement value at each follow-up was statistically significant compared to the baseline measurement (p<0.05). While the CD8+ T cell count decreased in the follow-up months compared to the baseline measurement, these changes were statistically significant because the measurements in the third and ninth months showed a significant decrease compared to the baseline measurement (p<0.05). Since the CD4/CD8 ratio increased compared to the baseline measurement and the eGFR value decreased compared to the baseline measurement, the measurement in each follow-up month was statistically significant compared to the baseline measurement (p<0.05). During follow-up, no statistically significant change was detected in lipid values compared to baseline.

## Discussion

Due to the success of ART in achieving virological suppression, PLWHs are now living longer and developing age-related comorbidities with specific therapeutic and monitoring needs. Success in HIV treatment is possible with drug regimens that could potentially increase patient compliance and have fewer side effects than current drugs. In this regard, BIC/FTC/TAF as a single-tablet regimen, which has been approved by the FDA for patients with low renal and/or bone toxicity, has become a preferred regimen recently in clinical studies due to its high virological suppression rates and low side effect profile [13,14]. In Turkey, studies on BIC/FTC/TAF treatments in real-life settings are limited. Following the approval of this treatment, our center was one of the first to adopt its clinical use in Turkey. We analyzed the use of BIC/FTC/TAF for a one-year period, and 56.8% of those who received BIC/FTC/TAF were treatment-experienced patients. It was determined that 93.9% of the previous treatment regimens of the experienced patients were EVG/c/FTC/TAF, and 4.5% of them used DTG+TDF/FTC. Those who received EVG/c/FTC/TAF were dependent on a medication change upon the recommendation of the physician after BIC/FTC/TAF was approved in our country. Treatment of those who had received DTG+TDF/FTC was changed to BIC/FTC/TDF due to headache in one person and rash in two patients. In other similar real-life studies, the reason for the change to BIC/FTC/TAF was the simplification of the treatment regimen, which was the most important reason for clinicians to initiate the transition [15,16]. Compared to published studies, the causes of switching treatment to BIC/FTC/TAF are different in our study, and this is one of the points that differentiates our study from other similar studies. This may be due to the high number of patients who previously used a single-tablet regimen in our clinic and the high rates of virological suppression among patients in treatment in our country [11].

Discontinuation of BIC/FTC/TAF was seen in four PLWHs. Rash and headache were adverse effects that resulted in the switch of the treatment regime. Central nervous system-related adverse effects, most commonly headache, are observed with this type of integrase inhibitor [17]. Nonetheless, during the study period, weight gain and fatigue were observed mostly among our patient group. Most studies with BIC/FTC/TAF have shown greater weight gain in the treatment-naive group than in the experienced group [18,19]. In our study, weight gain was found to be equal in the naive and treatment-experienced groups (12% versus 12.1%); no statistically significant difference was found (p=1.000) (data not shown). In a meta-analysis of INSTI use-related weight gain, it was found that DTG was the most common, followed by BIC, RAL, and EVG [20].

The clinical trials of BIC/FTC/TAF were reported to yield high virological responses. A randomized clinical meta-analysis of antiretroviral-naive and experienced PLWHs showed that BIC/FTC/TAF was effective at week 48 [18]. In another meta-analysis of five clinical trials, virological suppression rates with BIC/FTC/TAF at week 48 were found to be higher in people who were virologically suppressed at baseline (95%) and had no ART experience (87%), regardless of age [21].

Real-life studies of the efficacy of BIC/FTC/TAF have generally been conducted in treatment-experienced patients. In a study conducted in patients over 50 years of age with treatment experience, virological success was observed to continue [22]. A study in Spain showed that among treatment-experienced PLWHs who switched to BIC/FTC/TAF, undetectable HIV viral loads persisted at 48 weeks [23]. In our cohort, switching to BIC/FTC/TAF was associated with the maintenance of virological suppression at week 48. In half of the treatment-naive patients, HIV-RNA was <50 copies/mL in the first month of treatment, while the viral suppression rate was 100% in the ninth and 12th months.

In a real-life study from February to October 2021 from Romania, a total of 122 treatment-naive patients who started BIC/FTC/TAF displayed rapid immunological and virological response, with an increase to a median CD4+ T cell count of 320 cells/mm³ and a decrease in HIV viral load to a median of 156 copies/mL over the course of follow-up (the duration follow-up was not uniform) [16]. In another study of naive patients from large reference clinical centers, suppression rates for treatment-naive patients at six months were lower than 80% but higher at 12 months (92%) [24]. In our study, treatment-naive patients with HIV-1 RNA levels below 50 copies/mL were 55.9% in the first month, 73.7% in the third month, 90.2% in the sixth month, and 100% in the ninth and 12th months. Our study demonstrated good viral suppression in treatment-naive patients.

In our study, the median CD4+ T cell count in naive patients was 527 cells/mm^3^, with an increase to over 757 cells/mm^3^ at 12 months. After INSTI use, the one-year increase in CD+4 T cells should be modified by 200 cells/mm^3^ or more per year. It is known that the number of CD4+ T cells increases annually by 100-150 cells/mm^3^ on average [25]. In a meta-analysis of seven clinical studies, it was determined that the increase in the number of CD+4 T cells in the BIC/FTC/TAF arm was higher in treatment-naive patients than in experienced patients [18].

In the BIC/TAF/FTC switch study, the median CD4+ T cell count increased from 568 cells/L at baseline to 610 cells/L after 48 weeks of follow-up. A statistically significant increase in CD4+/CD8+ ratio was also detected, from 0.72 to 0.81 [26]. An observational study of a prospective cohort of ART-naive patients comparing two drugs versus three drugs found similar CD4/CD8 ratio recovery rates at 48 weeks between 2DR with DTG plus 3TC and 3DR based on DTG or BIC [27]. In our study, we observed that the CD4/CD8 ratio increased by 0.6 to 0.8 and 0.8 to 0.9 at week 48 compared to the baseline measurement in patients new to ART and treatment-experienced patients, respectively. BIC/FTC/TAF was successful in maintaining a near-perfect immunological efficacy in all patients.

Other clinical studies reported different results on serum cholesterol and triglyceride of BIC/FTC/TAF. The study by Maggiolo et al. showed significant reductions in total cholesterol and triglycerides among those who switched to B/FTC/TAF at week 48, with no significant change in low-density lipoprotein (LDL) cholesterol, high-density lipoprotein (HDL) cholesterol, or total cholesterol/HDL ratio [10].

In a study that analyzed five clinical trials, LDL/TC levels were increased by 2.7%/0.3% in the BIC/FTC/TAF treatment and 5.9%/2% in the comparator regimen [21]. A real-life study conducted with HIV patients over 50 years of age found a significant change in lipid parameters at 48 weeks. The median TC was decreased by 15 mg/dL, HDL was decreased by 1 mg/dL, LDL was decreased by 8 mg/dL, and triglycerides were decreased by 18 mg/dL. At the baseline, 179 (51%) patients were receiving lipid-lowering therapy; during the study, 42 (12%) patients were started on lipid-lowering therapy, and 11 (3%) patients discontinued the therapy [22]. In our study, we did not detect significant changes in lipid parameters with BIC/FTC/TAF in both treatment-experienced and treatment-naive patients.

In our study, baseline eGFR was found to be lower in the treatment-experienced patients than in the naive patients. No statistically significant difference was found in eGFR change in the treatment-experienced patients during the 48-week follow-up period. In the treatment-naive patients, baseline eGFR was higher than in the treatment-experienced patients, and it was statistically significantly lower during treatment, except for the third month. In the Bictegravir Single Tablet Regimen (BICSTaR) study, similar to our study, a statistically significant decrease in eGFR was found in treatment-naive patients at 12 months compared to baseline eGFR [28]. In the same study, a statistically significant decrease was also found in treatment-experienced patients. In a study comparing DTG/3TC and BIC/FTC/TAF, a decrease in eGFR was found in the DTG group. It was noted that patients in the DTG group had a decrease in eGFR, probably due to DTG's inhibition of organic cation transporter 2 (OCT2) and thus decreased tubular creatinine excretion. This effect was not observed in the BIC group [29]. In a switch study conducted in virally suppressed patients over 65 years of age, it was found that no patient developed proximal tubulopathy, and changes in renal biomarkers were consistent with the known renal safety profile of TAF [10]. The decrease observed in eGFR in our study may also be due to the effect of BIC known as an inhibitor of renal transporters, organic cation transporter 2 (OCT2), and multidrug and toxin extrusion 1 (MATE1) [8]. However, since treatment-experienced patients in this study received EVG/c/FTC/TAF before switching to BIC/FTC/TAF and cobicistat is also an inhibitor of cation transporters, predominantly MATE1, direct conclusions cannot be drawn about the effect of BIC on renal transporters in these patients [10]. Similarly, in our study, due to the high number of patients using EVG/c/FTC/TAF, there was no decrease in eGFR levels in the treatment-experienced patients.

The limitations of our study are that the analysis was retrospective, confounding factors could not be controlled, and data were obtained from a single center in Turkey, which limits generalizability to other populations.

## Conclusions

In our real-life observational study, BIC/FTC/TAF treatment achieved rapid viral suppression, maintained viral suppression in virally suppressed patients, and was effective for immunological recovery in both treatment-experienced and naive HIV patients. No serious adverse effects were observed. Our study has proved the potential of BIC/FTC/TAF as an important option in the treatment of HIV patients.
